# Mediastinal Teratoma Mimicking Neurofibroma in CT-Guided Biopsy in a Patient With Neurofibromatosis Type 1

**DOI:** 10.7759/cureus.36562

**Published:** 2023-03-23

**Authors:** Andreas Gkikas, Sofoklis Mitsos, Achilleas Antonopoulos, Nikolaos Korodimos, Elias Santaitidis, Nektarios Koufopoulos, Alina-Roxani Gouloumis, Periklis Tomos

**Affiliations:** 1 Thoracic Surgery, UCLH, London, GBR; 2 Thoracic Surgery, National and Kapodistrian University of Athens, “Attikon” University Hospital, Athens, GRC; 3 Pathology, Attikon University Hospital, Medical School of Athens, Athens, GRC; 4 Pathology, 2nd Department of Pathology, National and Kapodistrian University of Athens, “Attikon” University Hospital, Athens, GRC

**Keywords:** intrathoracic neurofibroma, neurofibromatosis type 1 (nf-1), neurofibromatosis 1, adult teratoma, anterior mediastinal mass, mediastinal germ cell tumor, mediastinal tumours, mediastinal neoplasms, mediastinal tumors, general thoracic surgery

## Abstract

Teratomas are a type of germ cell tumor that may contain several different types of tissue. Neurofibroma is a benign peripheral nerve sheath tumor with the plexiform type being pathognomonic for neurofibromatosis type 1. We report a case of a 33-year-old woman with a background of Neurofibromatosis type 1 who presented with left-sided chest pain and shortness of breath. She was diagnosed with a large mediastinal mass which was confirmed from a CT-guided biopsy as neurofibroma. Following a multidisciplinary team discussion, she underwent mediastinal mass resection and the final histopathology report revealed mediastinal mature teratoma.

## Introduction

Neurofibromatosis type 1 (NF1) is a hereditary disorder that is either inherited in an autosomal dominant pattern or by de novo mutations. It is caused by mutations or deletions to the NF1 gene which is located on chromosome 17 and encodes the tumor suppressor protein neurofibromin [1]. We herewith describe an extremely rare case of mediastinal mature teratoma in a young woman with NF1 which was mimicking neurofibroma at the initial biopsy. To the best of our knowledge, only two cases of retroperitoneal and one case of testicular teratoma have been reported in patients with NF1 [2-4].

## Case presentation

The patient was a 33-year-old Caucasian woman who presented with left-sided chest pain and shortness of breath which progressively worsened over 12 hours. She was experiencing intermittent and brief episodes of left-sided chest pain and dyspnoea over the last year which did not cause her any immediate concerns as they were self-terminating. During the same period, she noticed increasing hair loss throughout her body. Furthermore, she developed a subcutaneous nodule below her right breast which reached dimensions of 6.5 x 5 cm within a year. On clinical examination, she had reduced air entry on the left base during her chest auscultation, café-au-lait spots throughout her body, some larger than 15mm, and bilateral inguinal freckles. Her medical history was comprised of the excision of two subcutaneous nodules 10 years ago (located at the ulnar side of her left forearm and the posterior triangle of her right neck) which led to her NF1 diagnosis. 

**Figure 1:**
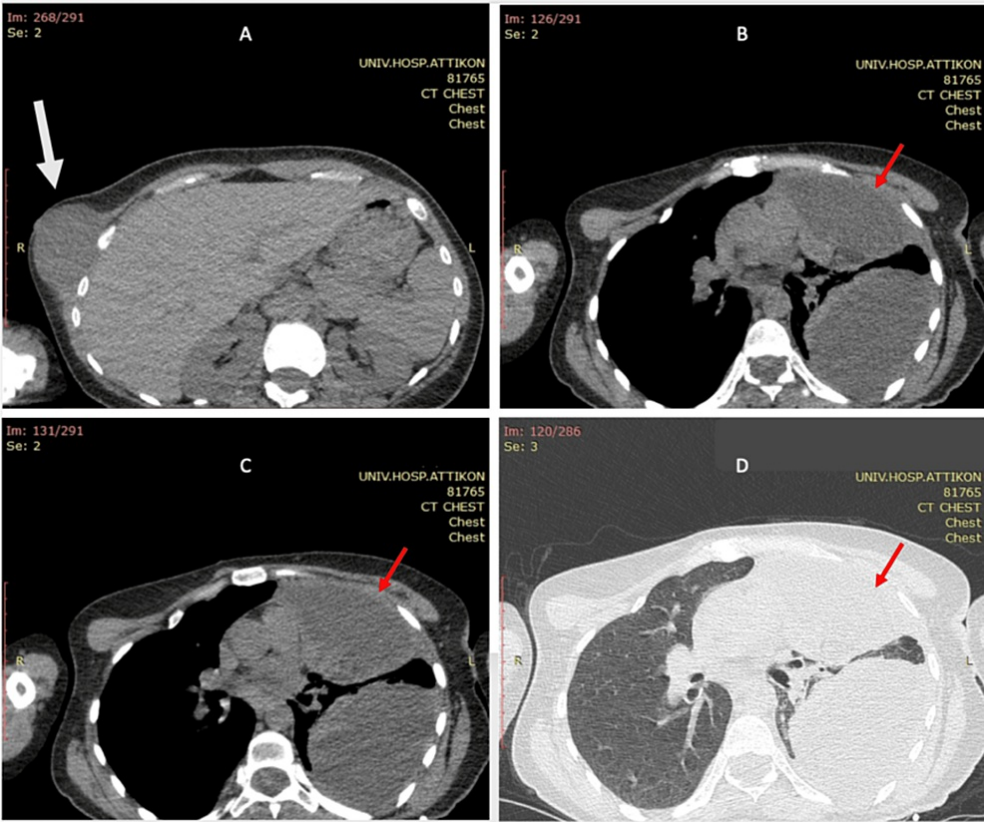
CT scan showing (A) well-defined hypodense large subcutaneous mass arising from the right anterior chest wall (grey arrow) and (B-D) large mediastinal mass anteriorly to the LUL with size 10.9 x 7.9 cm consistent with soft and fat tissue densities and thickening of its wall (red arrows). Increase in mediastinal shift to the right and small to moderate right sided pleural effusion. Presence of lesions with homogenous density in the right pleura with paravertebral location and widening of the right intravertebral spaces (T8-T10) indicating of a neuroma. Small atelectasis at the RLL, no pericardial effusion, lymph node enlargement in the mediastinum and axillary region. CT: Computer Tomography; LUL: Left upper lobe; RLL: Right lower lobe.

Thoracic imaging revealed a large left-sided pleural effusion and a large mass in the anterior mediastinum and therefore, she was subsequently referred to our department. The size of the mass on the computer tomography (CT scan was 10.9 x 7.9 cm with soft tissue and fat densities, and wall thickening, and it appeared to arise from the left phrenic nerve (Figures 1A-1D). While an inpatient, her pleural effusion was managed with a chest drain and her case was discussed in the local thoracic multidisciplinary team (MDT) meeting. In total, 2.4L of exudate was drained from her chest. Fluid cytology was in keeping with non-specific chronic inflammation showing only a scarce presence of neutrophils and no signs of malignancy. Her tumor markers were negative apart from a raised CA 19-9: 84.8 U/mL.

**Figure 2:**
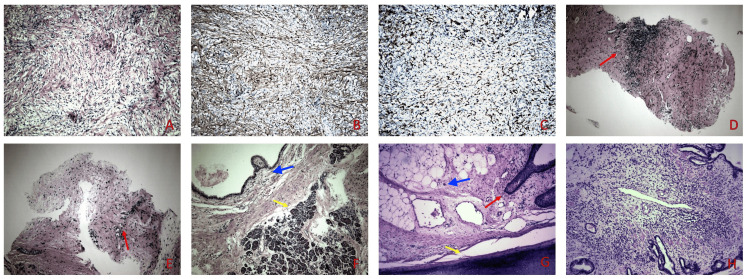
Histopathology. The incisional biopsy revealed a moderately cellular, spindle cell proliferation without significant atypia, or mitotic activity (2A). Immunohistochemistry showed positive staining for S-100 (2B) and CD34 (2C). The CT-guided biopsy consisted mainly of fibrous tissue with focal lymphocytic aggregates (red arrow) (2D). Small area with similar histological features with the previously diagnosed spindle cell proliferation of the incisional biopsy (red arrow) (2E). Histopathology of the surgical specimen showed predominantly mature teratoma with tissues from all three germ cell layers including respiratory type epithelium (blue arrow), pancreatic parenchyma (yellow arrow) (2E), skin (red arrow), hyaline cartilage (yellow arrow), and mature fat tissue (blue arrow) (2F). Area with immature mesenchyme adjacent to respiratory type epithelium (2G). CT: Computer Tomography.

The most prevalent diagnosis in our differential was that the two masses were the manifestation of the NF1 with a cutaneous neurofibroma at the chest wall and a plexiform neurofibroma arising from the left phrenic nerve. In order to define if there was any clinical correlation between the two masses, we performed histological examination. We proceeded with an incisional biopsy of the right subcutaneous mass from the anterior chest wall followed by a CT-guided biopsy of the anterior mediastinal mass. The incisional biopsy showed a moderately cellular, spindle cell proliferation without significant atypia, or mitotic activity (Figure 2A). Tumor cells stained for S-100 and CD34 (Figures 2B, 2C). The CT-guided biopsy consisted mainly of fibrous tissue with focal lymphocytic aggregates (Figure 2D) and few hemosiderin-laden macrophages. At the edge of the core there was an area which resembled morphologically with the previously diagnosed spindle cell proliferation of the incisional biopsy (Figure 2E). Despite low risk of malignancy from the biopsy results, the consensus from the thoracic MDT was to proceed with excision of the anterior mediastinal mass due to persistence of symptoms.

**Figure 3:**
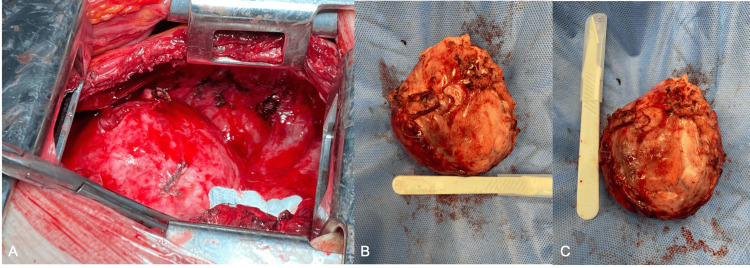
Intra-operating findings of mediastinal mass. (A) Smooth, lobulated mass arising from the anterior mediastinum seen through left posterolateral thoracotomy. (B, C) Resected anterior mediastinal mass sized 10.9 x 7.9 cm.

She underwent a left posterolateral thoracotomy and anterior mediastinal mass excision under single-lung ventilation. Upon surgical exploration of the mediastinum, the large mass in the anterior mediastinum was easily identified (Figure 3). It presented as a red mass with a smooth and lobulated surface with no adhesions to the pericardium, the lung parenchyma or the great vessels. Furthermore, the mass was in close proximity but did not appear intra-operatively to arise from the phrenic nerve as it was originally thought but it was pedunculated with a short stalk of less than 1cm from thymic fat in the anterior mediastinum. Tissue dissection and excision of the mass was performed with relative ease and it was sent for histopathology. A single chest drain was inserted and intra-operative intercostal blocks were performed using 20mL of 0.5% levobupivacaine. Following absence of air leak and output of 150mL over 24 hours, the chest drain was removed on the first postoperative day and the patient was discharged home on postoperative day 5 following adequate pain control and respiratory physiotherapy. She was discharged with 1g of paracetamol four times a day along with additional doses of 400mg ibuprofen when required up to three times a day.

The histopathology report revealed a predominantly mature teratoma which included tissues from all three germ cell layers (skin, hyaline cartilage, mature fat tissue, pancreatic parenchyma and respiratory type epithelium) (Figures 2F, 2G). Focally, an area with immature mesenchyme adjacent to respiratory type epithelium was found (Figure 2H). Immature neuroepithelial tissue or malignant transformation were not observed and resection margins were tumor free. Since then, she has been scheduled for six-month interval follow-up visits with thoracic imaging with no signs of disease recurrence at her first outpatient appointment.

## Discussion

Benign teratomas in the mediastinum are a rare diagnosis that accounts for 3%-12% of mediastinal tumors [5]. However, they remain the most prevalent germ cell tumors in the mediastinum [4]. The majority of mediastinal teratomas are slow growing and are either diagnosed incidentally after thoracic imaging or because they provoke symptoms due to compression to surrounding tissues. In compliance with the literature, our patient also presented with left-sided chest pain and shortness of breath which resulted because of the mass effect of the tumor on the anterior mediastinum. The elevated CA 19-9 levels in our case could be attributed to the presence of pancreatic cells and bronchial mucosa in the teratoma as reported in previous cases of teratoma without this being considered pathognomonic [6,7].

NF1 is a rare disease with an incidence of approximately 1:3,000 people [8]. Patients diagnosed with the disease are born with an autosomal dominant mutation on the tumor suppressor gene *NF1* that encodes neurofibromin. This is a protein that acts as a GTPase that down-regulates the RAS pathway [9]. The pathogenesis, intracellular pathways, and even cell of origin that result in the formation of neurofibromas have been widely debated [10]. The most prominent cellular lineage for the development of plexiform neurofibromas has been the Schwann cells. However, the exact point in their development from neural crest stem cells to mature myelinating or non-myelinating Schwann cells is a topic of ongoing research. Similarly, details in the pathogenesis and cell origin of the cutaneous neurofibromas also remain unanswered [10]. Evidence suggests that cutaneous neurofibromas arise from a stem cell population, which is found in the skin dermis, and known as skin-derived precursors. However, that consists of a heterogeneous cell group that is multipotent and can differentiate into Schwann cells, adipose tissue, or neurons [11]. Therefore, the specific cell subtype which in absence of *NF1*, results in the formation of neurofibromas has not yet been clearly defined.

Mature teratomas are derived from all three embryonal germ cell layers [12]. The anterior mediastinum has been reported as the most frequent extra-gonadal location of these tumors. CT chest is considered to be the radiographic modality of choice for their diagnostic evaluation with the presence of teeth and/or fat-fluid level considered pathognomonic [13,14]. They present with heterogenous characteristics which extend from sharp margins to well-rounded or lobulated masses with the presence of soft tissue, fluid, fat, or calcium attenuation.

In our patient, the CT scan revealed a lobulated mass with soft and fat tissue densities with no evidence of teeth which would have supported the diagnosis of mature teratoma preoperatively. In compliance with the histological criteria for fibroadenoma diagnosis, the incisional biopsy from the chest wall mass in our patient demonstrated spindle cell proliferation with positive staining for S-100 and CD34 (Figure 2A-2C) [15-17]. Furthermore, the results from the CT-guided core biopsy of the mediastinal mass demonstrated a spindle cell proliferation at its edge which appeared morphologically similar to that of the incisional biopsy of the chest wall mass. Therefore, according to the results of our pre-operative investigations and our patient's past medical history of NF1, the diagnosis of a plexiform fibroadenoma was considered to be the most prevalent.

To our knowledge, this is the first case of teratoma diagnosis in the anterior mediastinum in a patient with NF1. Anterior mediastinal tumors in NF1 patients are usually either phrenic neurofibromas or malignant peripheral nerve sheath tumors (MPNSTs) which are rare sarcomas [18,19]. Only nine case reports of phrenic neurofibromas have been reported and less than 20 cases of MPNSTs with NF1 being the most important risk factor [18,19].

Furthermore, apart from a unique diagnosis our report also presents an additional valuable feature for the medical literature, as the initial CT-guided biopsy of the anterior mediastinal mass was not in agreement with the final histopathology.

## Conclusions

Neurofibromatosis type 1 is a rare autosomal disease. Its most commonly reported clinical manifestation of the anterior mediastinum is either phrenic neurofibromas or malignant peripheral nerve sheath tumors. The patient in our study, despite she had also developed subcutaneous neurofibromas, her mediastinal mass was confirmed as predominantly mature teratoma. This case report aims not only to highlight the first case of teratoma diagnosis in the anterior mediastinum among patients with NF1 but also to raise awareness among clinicians that a mature teratoma could mimic neurofibroma in a CT-guided biopsy.
